# Appendiceal Vaginal Fistula: A Rare Complication of Nonoperative Appendicitis Management

**DOI:** 10.7759/cureus.49699

**Published:** 2023-11-30

**Authors:** Madison Brune, Milot Thaqi, Kevin Bartow

**Affiliations:** 1 Surgery, University of Missouri School of Medicine, Columbia, USA

**Keywords:** appendico-vaginal fistula, nonoperative treatment, antibiotics, laparoscopic appendectomy, appendicitis treatment, vaginal fistula

## Abstract

Appendicitis is one of the most common conditions encountered in emergency surgical practice. An appendico-cutaneous fistula is a rare complication of appendicitis. An appendico-vaginal fistula is extremely rare. To our knowledge, based on a thorough review of the literature using PubMed, MEDLINE, and Google Scholar, only three other cases of an appendico-vaginal fistula have been reported. We present one such case in a 43-year-old female with a history of partial hysterectomy, recurrent abscesses that had failed to respond to repeated drainage and antibiotic treatment, and nonoperative treatment of appendicitis.

## Introduction

Appendicitis is one of the most common causes of acute abdominal pain worldwide, with a lifetime risk of 7% to 8% [1]. Appendicitis is an inflammation of the appendix, a small pouch-like structure attached to the beginning of the large intestine. The condition can manifest in varying degrees of severity, often classified as early, late, and chronic forms. Early appendicitis typically presents with vague abdominal pain that starts near the navel and then shifts to the lower right quadrant, accompanied by nausea, vomiting, and a decreased appetite. As the inflammation progresses, the symptoms intensify, and the patient might experience sharp, localized pain, fever, and an elevated white blood cell count [2]. If not treated promptly, late-stage appendicitis can ensue, characterized by pronounced symptoms including severe abdominal pain, rigidity in the right lower quadrant due to peritonitis, and high fever [3]. Chronic appendicitis, on the other hand, is a relatively rare condition wherein patients experience recurrent abdominal pain over the course of weeks to years. It can sometimes be challenging to diagnose due to its intermittent and milder symptoms [4]. Complications from untreated or inadequately treated appendicitis include the development of an abscess, generalized peritonitis, or sepsis, all of which can be life-threatening [5].

Fistula formation from the appendix into adjacent organs is rare, most commonly involving the bladder, skin, and ileum. The occurrence of an appendiceal vaginal fistula is extremely rare. Only three cases have been reported. A systematic review of the literature evaluating all appendiceal fistulas reports the relative frequency of appendiovaginal fistula formation to be 1.2% [1]. In this article, we report a case of appendiceal vaginal fistula formation associated with appendicitis following nonoperative management.

## Case presentation

A 43-year-old female with a past medical history significant for type I diabetes mellitus, tobacco use, and a history of partial hysterectomy for cervical cancer initially presented to an outside facility. At that time, the patient was diagnosed with perforated appendicitis and was managed with percutaneous drain placement and antibiotics. The patient presented six months after with a recurrence of her abdominal pain following accidental drain removal and febrile episodes. Imaging demonstrated persistent abscess formation measuring 6 cm in the largest dimension. The decision was made to proceed with percutaneous drain placement and antibiotics, as the patient was reluctant to proceed with surgery. At this time, the drain output was between 5 and 15 cc/day typically serosanguineous in appearance.

**Figure 1:**
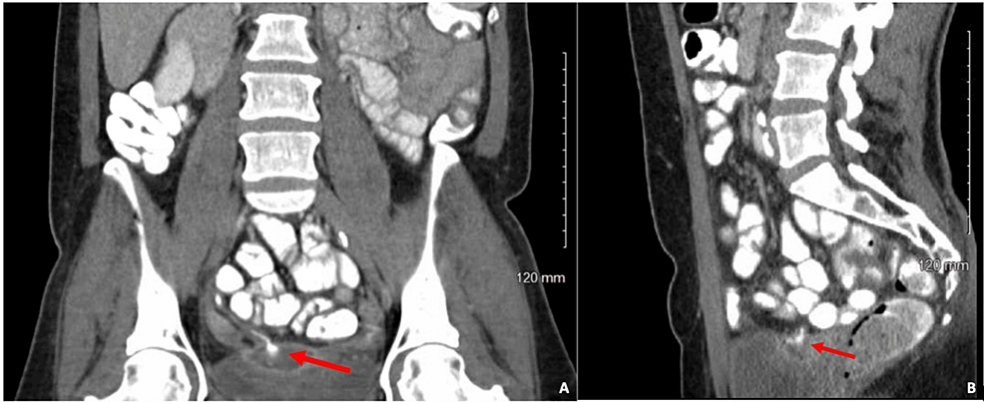
Appendico-vaginal fistula indicated by the red arrows

The patient was seen in the clinic two months later, with improvements in her symptoms, and the drain was removed. In total, the drain was in place for nine months. The patient ultimately re-presented to the hospital one month after with increased abdominal pain, vaginal discharge, recurrent pelvic abscess on imaging, and continued reluctance to proceed with surgery. A decision was made to proceed with repeat percutaneous drain placement and antibiotics therapy. The patient was subsequently lost to follow-up; however, the patient had presented to an outside facility for drainage removal and treatment of urinary tract infections and vaginal discharge on numerous occasions. The patient ultimately re-presented to our institution eight months later, with imaging consistent with an appendiceal vaginal fistula, with passage of foul-smelling discharge. CT scan of the abdomen and pelvis was performed with oral, rectal, and IV contrast media. The contrast CT scan showed a 4.7 x 2.0 x 2.0 cm abscess contiguous with the appendix and evidence of an appendiceal vaginal fistula (Figures 1A, 1B).

The decision was made to proceed with laparoscopic appendectomy, fistula takedown, drainage of intra-abdominal abscess, and pedicle omental flap. The postoperative period was uneventful, and the patient was discharged home on postoperative day two. The pathology demonstrated an appendix without any acute appendicitis or evidence of malignancy.

## Discussion

Acute appendicitis is the most common reason for emergency abdominal surgery [5]. Anatomically, the appendix is a blind-ending structure. Direct luminal obstruction can lead to infection and inflammation, eventually leading to appendiceal perforation and discharge of the luminal contents [6]. Historically, appendectomy has been the standard treatment for acute appendicitis regardless of presentation [7]. Recent clinical trials have suggested that antibiotic therapy is not inferior to surgery for uncomplicated appendicitis in healthy adults [8,9]. However, appendectomy remains the standard of care for acute appendicitis [10]. Nonoperative management also plays a role in the case of complicated appendicitis. Stable patients with localized symptoms such as an abscess less than 3 cm should be managed with immediate appendectomy. Abscesses greater than 3 cm should be drained percutaneously and antibiotics should be given. If clinical improvement occurs, then discharge on antibiotics and follow-up with appendectomy six to eight weeks later is appropriate [11].

If left untreated, appendicitis can result in complications such as rupture, peritonitis, sepsis, and possible death [5,12]. Rare complications include the formation of an appendiceal fistula, which can be described as 'the primary perforation of the appendix to an adjacent hollow viscus or to the skin’ [13]. Many cases of appendicular fistulas have been reported, including appendico-vesical, appendico-cutaneous, and appendico-ileal fistulas [1]. However, appendico-vaginal fistulas are far less common, potentially due to the smaller probability of an anatomic encounter [14,15].

Appendico-vaginal fistula formation occurs when the appendix comes into direct contact with the vagina and ruptures forming a fistula [14]. The fistula tract may persist due to epithelization. Based on a systematic review evaluating reports of fistulae involving the appendix, the most common etiology of appendico-vaginal fistulas in past cases is appendiceal carcinoma followed by prior hysterectomy [1]. Based on the pathology report, appendiceal carcinoma is not likely to be the underlying etiology of the appendico-vaginal fistula in our patient. Whether the patient's previous partial hysterectomy was related is uncertain. However, it stands to reason that adhesions from the hysterectomy may have made it possible for the appendix to come into closer proximity to the vaginal cuff [15]. Furthermore, repeat percutaneous drain placement increases the risk of injury to the pelvic structures, including the vagina, and may have allowed a fistula to form [14,16].

Diagnosis of an appendico-vaginal fistula is often difficult. Most patients present with foul-smelling vaginal discharge and are treated as urinary tract infections [17]. The patient's clinical signs and symptoms can be masked due to antibiotic treatment. CT scan with oral contrast of the abdomen and pelvis is the standard imaging modality to assess for an appendiceal fistula. Oral contrast may be seen in the vaginal vault delineating the fistula tract and assisting the surgeon in operative planning. The procedure of choice includes appendectomy followed by vaginal vault repair. While appendico-vaginal fistulas are rare, patients with predisposing factors, such as radiation [17], appendicular diverticulitis, Crohn’s disease [18], carcinoid tumors, neuroma [19], cystic fibrosis [20], and cystadenocarcinoma of the appendix [21], may be at increased risk for fistula formation.

## Conclusions

We present a case of an appendiceal vaginal fistula formation following nonoperative management of perforated appendicitis. While more literature demonstrates the noninferiority of medical management of acute appendicitis, the risk of persistent abscess and fistula formation is of concern. It should be discussed with patients wishing to proceed with medical management.
